# Implementation of a virtual dementia system of care in a VA health setting

**DOI:** 10.1186/s12877-025-06034-0

**Published:** 2025-05-28

**Authors:** James S. Powers

**Affiliations:** Geriatric Research Education and Clinical Center, Tennessee Valley Healthcare System, Nashville, TN 37212 USA

**Keywords:** Alzhiemer’s disease, Dementia care, Caregivers

## Abstract

**Objective:**

Dementia care remains supportive and geriatric resources are scarce. We describe a dementia system of care consisting of virtual and e-consults working through the primary care provider, as well as providing virtual group caregiver support. These models were developed based on patient and caregiver preferences and necessitated by the Covid public health emergency.

**Methods:**

A geriatrician supported dementia consult clinic transitioned to a virtual model of care. A centrally located caregiver support group providing a 4-week curriculum and delivered in conjunction with a social worker similarly transitioned to virtual support. Primary care providers generated consults and were provided educational consultations regarding dementia diagnoses and management in the primary care setting.

**Results:**

Between 2018 and 2024, 1176 consultations were provided during this period as the clinic transitioned from in-person to virtual consultation. Recommendation categories included: (1) diagnostic testing, (2) medication recommendations and deprescribing, (3) referral for formal neuropsychological testing, (4) psychiatric referral for behavioral concerns, (5) primary care management and goals of care, (6) safety considerations, (7) home and community-based services, and (8) caregiver support. Providers continue to send new consultations and request follow-up advice on previous consults. A total of 72 family caregivers participated in a virtual 4-class support curriculum.

**Conclusions/Impact:**

Virtual and e-consult dementia care working through the primary care provider, as well as virtual group caregiver support, are feasible, acceptable and sustainable models of dementia care to efficiently utilize scarce geriatrics resources serving a wide geographic area. A virtual dementia system of care may facilitate PCP delivery of supportive care for persons living with dementia, dementia care navigation, and caregiver support.

## Introduction

Care for persons living with dementia (PLWD) is challenging and the majority of patients with Alzheimer’s Disease are supportively managed in the primary care setting, with 85% of dementia diagnoses provided by primary care providers [1]. The number of PLWD over age 65 is estimated at 6.9 million and is expected to rise to 13.8 million by 2060, [2] creating a burden for primary care providers, as well as an estimated 11 million unpaid family caregivers [2]. There is an inadequate number of specialty providers in Geriatrics, Neurology and Psychiatry to evaluate and manage all PLWD. Primary care providers have a relationship with patients and families and are in a good position to provide supportive care to PLWD and their caregivers, however they may need assistance in this role due to the to the complexities involved in management. Telehealth has been found to enhance access to care and to be effective for behavioral health, education, and caregiver support [3–6]. We report a 7-year experience (2018–2024) developing and transitioning to a virtual dementia system of care in a VA health setting.

## Methods

The Department of Veterans Affairs Tennessee Valley Healthcare System (TVHS), is a health care system with 140,000 patients in Middle Tennessee. There are 2 campuses located 40 miles apart, with 20 affiliated community-based outpatient clinics (CBOC’s). An estimated 8,500 dementia patients are cared for at TVHS.

In 2011, TVHS developed a geriatric patient-centered medical home model, the geriatric patient-aligned care team (GeriPACT) [7]. GeriPACT is a special population PACT within primary care for complex geriatric and other high-risk vulnerable veterans providing integrated, interdisciplinary assessment and longitudinal management, and coordination. The practice is supported by the Department of Veterans Affairs Computerized Patients Record System (CPRS), including the electronic patient portal, *My* health*e*vet, with telemedicine capabilities. The TVHS population group from 100,000 to 140,000 over a 10-year period.

Since 2018, a geriatrician affiliated with GeriPACT, performed the majority of the dementia consults separate from the primary care geriatrics patient panel care, working through the referring PCP in primary care practices and CBOC’s. Neurology does not have a cognitive clinic at TVHS and limits primary cognitive disorder patients unless also associated with TBI or movement disorders. Psychiatry sees dementia patients with behavioral concerns.

Dementia consultation was initially conceived as an in-person consultation and management service provided by GeriPACT. Access concerns for referral patients led to the development of telehealth capability in 2018 and the dementia clinic transitioned to virtual care in response to the Covid 19 pandemic in 2020. Referrals from primary care providers were often triggered by dementia warning signs such as delirium with acute illness, wondering, self-neglect, need for appointment of a fiduciary, and caregiver concerns about behavioral manifestations and need for support services.

Consults were received electronically, responded to within 48 h, and consisted of a thorough review of the electronic health record and provision of an individualized educational consultation to the primary care provider with specific diagnostic and management recommendations. Primary care providers were requested to perform on-site diagnostic testing when indicated (i.e. thyroid function tests, B12, neuroimaging) and mental status screening. The SLUMS, [8] is preferred at VA as other validated tests such as the MMSE [9] and MOCA [10] have recently been monetized.

In conjunction with the dementia consult clinic, a virtual caregiver support group was initiated, co-facilitated by the geriatrician and Caregiver Support Program social worker [11]. A 4-class weekly curriculum was offered to caregivers and focused on emotional support, caregiving skills and sharing of best practices, as well as generating clinical referrals and supplying equipment needs for veterans. Similar to the dementia clinic, the caregiver support group became entirely virtual in 2020. Caregivers were referred to the support group by primary care providers as well as nurses and social workers caring for PLWD in other settings.

### Ethical approval and consent to participate

This study adhered to the Declaration of Helsinki. The Department of Veterans Affairs, Tennessee Valley Healthcare System (TVHS) Institutional Review Board (IRB) has determined this study as a quality improvement initiative and waived informed consent for participants.

## Results

**Figure 1:**
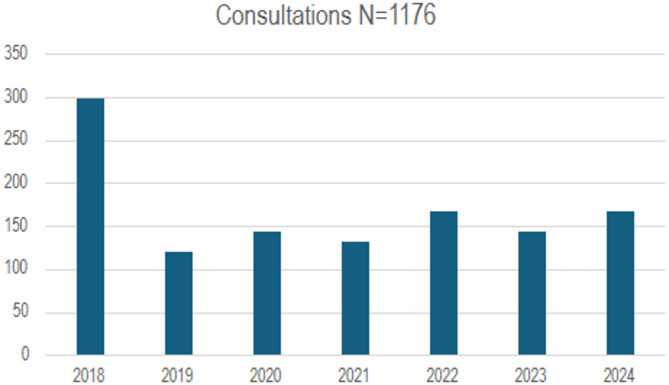
Fig. 1

Between 2018 and 2024, 1176 consultations were provided during this period as the clinic transitioned from in-person to virtual consultation. The service performed a mean 14 consultations monthly with no change pre-and post-Covid (t = 1.2, *p* =.3). (Fig. 1).

While the majority of consultations (93%) were e-consults, occasional (1%) telephone or video telehealth (veterans video connect VVC) calls with patients, in person visits, or telephone contact with providers was provided as requested or when more information was required. Some 5% of consultations were follow-up management questions from PCP’s.

Educational consultation responses with diagnostic information including functional assessment staging (FAST [12]) and specific management recommendations were provided to the referring clinicians including: (1) further diagnostic testing as individually indicated, (2) medication recommendations and deprescribing, (3) referral for formal neuropsychological testing when appropriate, (4) psychiatric referral for severe behavioral concerns, (5) individualized primary care management and goals of care, (6) safety considerations, (7) home and community-based services/social work involvement, and (8) caregiver support. Providers continue to send new consultations and request follow-up advice concerning previous consults, and family caregivers share contact information with each other during the group support sessions.

Acceptance of the virtual consultation by PCP’s remains high as indicated by these anecdotal comments:

*Appreciate the immediate access and support provided by the consultation.*

*Value the assistance with management of this difficult condition.*

A total of 72 family caregivers participated in the virtual 4-class support curriculum. Caregivers were 95% women with a mean age of 68. Many shared contact information with each other during the group support sessions, and voiced appreciation for the accessibility and support provided by the classes:

*Encouraged me to be a better caregiver! To be stronger! Always try to be more patient.*

*It helped me to know*,* I was not the only one that needed a break*,* or just to get outside and enjoy nature for a little while! To relax!*

*Would never have been able to attend in-person classes due to caregiving duties and travel distance.*

## Discussion

Telehealth, including e-consults and virtual consultation, improves access, decreases no-show rates, serves to educate PCP’s [13] and can combine to enhance the caregiver experience of care [11]. Anecdotal comments from patients and caregivers reflect acceptance of virtual care with enhanced access, efficiency, and effectiveness.

Enhancements to the e-consult/virtual model for dementia care could include tailored caregiver assessment and referral (TCARE), an evidence-based software used by federal, state, and private-funded agencies to identify caregivers at risk for burnout, connect them to the right services, and measure the impact of their programs [14]. TCARE partners with Area Agencies on Aging and community partners to deliver its caregiver support platform to communities of caregivers. Additionally, the GUIDE (Guiding an Improved Dementia Experience) service delivery mode is a national plan to address Alzheimer’s disease through a comprehensive package of care coordination and care management including caregiver education and support [15].

There remains a large need for screening strategies for appropriate candidates for new treatments, care navigation, and support services for caregivers and PLWD. For individuals receiving newer modalities of treatment including monoclonal antibodies directed at amyloid, there is also a great need for support services for caregivers as well as PLWD.

## Limitations

This model was developed in a VA care system and may not be applicable to all healthcare systems. A virtual dementia diagnosis may not appropriate for all patients such as those with severe behavioral disturbances and comorbidities including polytrauma, psychiatric disorders and PTSD. These patients may require formal neuropsychological testing. Additionally, some patient and caregiver concerns may not be able to be addressed by the PCP such as those with severe behavioral concerns or for whom the goals of care includes consideration of anti-amyloid treatment. Finally, telehealth and virtual educational consultation for dementia care require a skilled consultant familiar with primary care and knowledgeable about VA and other available home and community-based resources.

## Conclusion

While we embrace future advancements in the diagnosis and treatment of dementia, there is a large unmet need for continued supportive care for PLWD, dementia care navigation, and caregiver support. Virtual primary dementia care is feasible, acceptable, and effective and may help to address this need. A virtual dementia system of care may facilitate PCP delivery of supportive care for PLWD, dementia care navigation, and caregiver support.

## Data Availability

All data included in this study are available by request from the author.
